# Hydrophobic Interactions of Modified Coconut Oil and Pluronic 127 Enable Stable Formation of Bioactive Hydrogel for Onychomycosis

**DOI:** 10.3390/gels11080592

**Published:** 2025-07-31

**Authors:** Daniel P. Fitzpatrick, Grace Lawler, Carmel Kealey, Damien Brady, Jim Roche

**Affiliations:** 1 PRISM Institute, Technological University of the Shannon, N37 HD68 Athlone, Westmeath, Ireland; 2 Bioscience Research Institute, Technological University of the Shannon, N37 HD68 Athlone, Westmeath, Ireland; 3 Department of Pharmaceutical Sciences and Biotechnology, Technological University of the Shannon, N37 HD68 Athlone, Westmeath, Ireland; 4 Applied Polymer Technologies (APT), Technological University of the Shannon, N37 HD68 Athlone, Westmeath, Ireland; 5 Department of Applied Science, South East Technological University, R93 V960 Carlow, Carlow, Ireland

**Keywords:** antimicrobial, bioresorbable, coconut oil, drug delivery, hydrogel, wound regeneration

## Abstract

Fungal infections pose a significant yet under-recognised global health burden, affecting over one billion individuals annually and contributing to approximately 2.5 million direct deaths. The World Health Organisation (WHO) has recently reemphasised this issue through the publication of its Fungal Priority Pathogens List (FPPL) and its 2025 report evaluating current antifungal diagnostics and therapeutics. Among the most prevalent fungal pathogens is *Trichophyton rubrum*, an anthropophilic dermatophyte responsible for up to 70% of superficial fungal infections, including onychomycosis. The emergence of antifungal resistance further complicates management, necessitating the development of novel, effective, and sustainable treatment alternatives. Natural compounds are increasingly being explored for their antifungal potential due to their broad-spectrum activity and lower toxicity. Coconut oil has gained particular attention for its therapeutic properties attributed to medium-chain fatty acids (MCFAs), especially lauric acid. The aim of this study was to understand how innate and modified coconut oils can alter the rheological properties of Pluronic hydrogels while retaining antifungal activity for downstream application in treating fungal infections. Results identified hydrophobic interactions by FTIR and DSC between the hydrocarbon chains of the coconut triglycerides and the hydrophobic core of the Pluronic micelles, leading to gel stabilisation as identified by rheological analysis.

## 1. Introduction

Fungal infections represent a major global challenge, affecting both human health and agricultural productivity. Invasive fungal infections are most commonly seen in immunocompromised individuals, with a higher incidence observed in males [1], as well as in certain racial and ethnic groups [2]. Social determinants of health and existing health disparities play a significant role in increasing patient vulnerability to these infections [3]. Moreover, the growing population of immunosuppressed individuals introduces new risk factors and longer periods of susceptibility, presenting an ongoing challenge for healthcare providers [4].

Onychomycosis is a chronic fungal infection of the nail apparatus, primarily caused by dermatophytes such as *Trichophyton rubrum* and *Trichophyton interdigitale*, although non-dermatophyte moulds and yeasts, particularly *Candida* species, can also be implicated. The condition is characterised by nail discolouration, thickening, and onycholysis, often resulting in pain, discomfort, and significant cosmetic concerns. Onychomycosis accounts for up to 50% of all nail disorders and remains notoriously difficult to treat due to the dense keratin structure of the nail plate, which acts as a formidable barrier to drug penetration. *Trichophyton rubrum* (*T. rubrum*) is a filamentous, anthropophilic fungus with a strong affinity for keratinised tissues, including skin, hair, and nails. Its pathogenicity is largely attributed to a suite of virulence factors such as acid proteinases, elastase, keratinases, and other protein-degrading enzymes that support fungal adhesion, germination, penetration, and colonisation of the host [5]. *T. rubrum* is widely recognised as the primary causative agent of onychomycosis, particularly in toenails, followed closely by *T. mentagrophytes*, including its clonal variant *T. interdigitale*. Together, these two dermatophyte species are responsible for approximately 90% of toenail infections and 75% of fingernail infections [6]. *Candida albicans* is a unicellular yeast that is part of the normal human microbiota, commonly found in the mouth, gastrointestinal tract, and vaginal mucosa. While typically harmless, it can become opportunistic and cause infections (candidiasis) when the immune system is weakened or the microbial balance is disrupted [7].

The standard treatment for dermatophytosis primarily relies on oral or topical antifungal agents. However, unlike antibacterial treatments, the range of available antifungals is limited, and the pharmaceutical pipeline for new antifungal agents has slowed. In the past two decades, few novel antifungal drug classes have been introduced, despite rising resistance rates and an increasing population at risk [8]. The clinical challenge entails the identification of durable treatments for *T. rubrum* infections occurring in the distal and lateral subungual region of the human nail. Additionally, the availability of a limited repertoire of antifungal therapies, combined with their extensive utilisation, has played a role in the emergence of antifungal resistance [9,10,11].

Coconut oil is a lipid-rich substance composed primarily of saturated fatty acids. Coconut, harvested from the *Cocos nucifera* palm, is a major tropical crop cultivated across more than 90 countries, covering approximately 13 million hectares and producing over 62 million metric tonnes annually [12]. Belonging to the *Arecaceae* family, it is often referred to as the “tree of life” due to its extensive nutritional, medicinal, and economic value. Coconut oil is typically extracted from the dried kernel of mature coconuts, and its fundamental chemical composition was first characterised in the early 20th century [13]. Among its derivatives, virgin coconut oil (VCO) has gained widespread attention for its diverse therapeutic properties. It is particularly rich in MCFAs, primarily lauric acid, which are responsible for a broad spectrum of biological activities, including antioxidant, anti-inflammatory, immunomodulatory, hepatoprotective, wound-healing, and antimicrobial effects [14,15].

Pluronic^®^ F127 (PF127) is an FDA-approved, non-toxic triblock copolymer made of two hydrophilic poly(ethylene oxide) blocks and one hydrophobic poly(propylene oxide) block placed in the middle of the hydrophilic segments. Hydrophobic interactions play a central role in the self-assembly, stability, and performance of amphiphilic systems such as Pluronic-based hydrogels. These interactions arise from the tendency of nonpolar molecules or moieties to avoid contact with water, instead aggregating to minimise interfacial energy. To date, only polysaccharides have been exclusively investigated to induce this effect on Pluronic-based hydrogel systems as they contain ideal functional groups (amino (–NH2), carboxyl (–COOH), aldehyde (–CHO), and hydroxyl (–OH)), which are involved in hydrophobic interactions and warrant the formation of physical networks [16,17].

This article is the first to investigate how innate and modified coconut oils can alter the rheological properties of Pluronic hydrogels while retaining antifungal activity for downstream application in treating fungal infections.

## 2. Results and Discussion

### 2.1. Attenuated Total Reflectance Fourier Transform Infrared Spectroscopy (ATR-FTIR)

**Figure 1:**
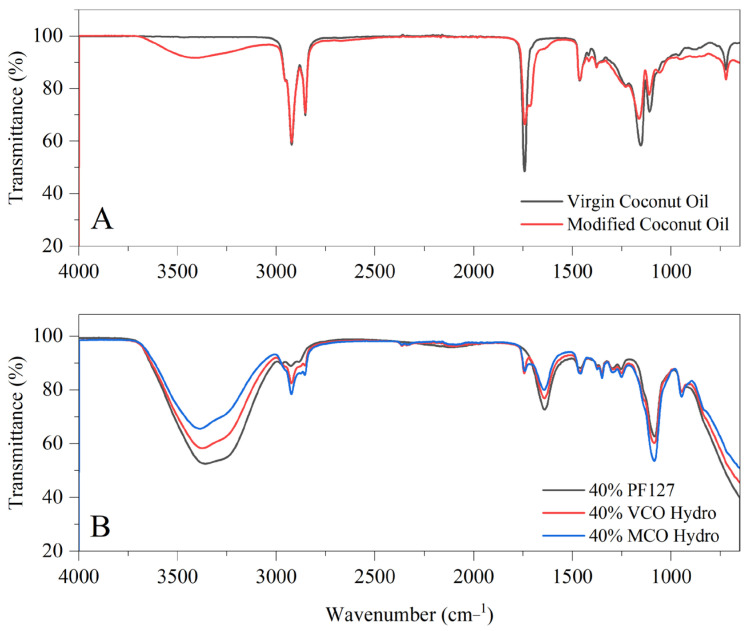
(**A**) FTIR spectra of innate virgin coconut oil (VCO) and modified coconut oil (MCO) and (**B**) FTIR spectra of 40% coconut oil-incorporated hydrogels.

In Figure 1, the FTIR spectra illustrating two key aspects: (A) the structural characteristics of virgin coconut oil and modified coconut oil, and (B) the variation in chemical composition upon incorporating these oils into PF127-based hydrogel systems. The innate VCO spectrum presents characteristic absorption bands consistent with its triglyceride rich composition, including prominent C–H stretching vibrations at 2920 cm^−1^ and 2850 cm^−1^, as well as a strong carbonyl (C=O) stretching peak near 1740 cm^−1^. Upon modification, the MCO spectrum displays several notable changes. A broader and more intense absorption band around 3400 cm^−1^ suggests a higher concentration of hydroxyl (–OH) groups, indicative of the introduction of polar functionalities. Additionally, subtle shifts and sharper features in the C–H stretching region point to alterations in the aliphatic chain structures. The fingerprint region (1500–1000 cm^−1^) reveals further distinctions, with MCO showing increased peak intensities and slight shifts, particularly around 1460 cm^−1^, 1370 cm^−1^, and within the C–O stretching region (1200–1000 cm^−1^), signifying structural modifications and new functional group contributions.

With respect to the oil-incorporated hydrogels, the FTIR spectra (B) highlights all hydrogel samples exhibited broad O–H stretching bands near 3400 cm^−1^, reflecting the increased hydroxyl content from the modified oil. C–H stretching peaks around 2920 cm^−1^ and 2850 cm^−1^ are observed in all formulations but are more pronounced in the VCO and MCO hydrogels than in PF127 alone, confirming the integration of lipid-based components. Importantly, a distinct peak near 1750 cm^−1^, attributed to ester carbonyl (C=O) stretching, appears in both VCO and MCO hydrogels, while absent in the innate PF127 hydrogel—denoting the presence of triglyceride structures within the oil-incorporated hydrogels. Further variations in the fingerprint region (1500–1000 cm^−1^), particularly in the C–O and C–C stretching regions, are most notable in the MCO hydrogel, which shows sharper and slightly shifted bands. These findings suggest that the modified oil not only retains key structural features but also interacts more extensively with the polymer network, potentially enhancing the chemical integration and functional performance of the hydrogel system. These interactions are further supported by the enhanced hydrophobic compatibility between the coconut oils and the Pluronic F127 matrix. As previous noted by Lupu et al. (2023), the nonpolar chains of both additives favour association with the hydrophobic polypropylene oxide (PPO) core of PF127 micelles, driven by hydrophobic interactions [16]. However, the presence of additional hydroxyl and carbonyl groups in MCO enables better alignment at the hydrophilic–hydrophobic interface of the micelles. This amphiphilic balance likely facilitates stronger interfacial adhesion and improved dispersion of MCO within the hydrogel, as suggested by the more defined FTIR peaks and enhanced spectral integration observed in MCO systems.

### 2.2. Differential Scanning Calorimetry

The differential scanning calorimetry (DSC) thermograms in the provided image reveal significant differences in the thermal behaviour and melt profiles of virgin coconut oil (VCO) and modified coconut oil (MCO) (A), as well as the thermoresponsive characteristics of PF127 and its derived hydrogel formulations incorporating either VCO or MCO (B). These observations provide an understanding into the physicochemical stability and thermal transitions of the bioactive formulations intended for treating onychomycosis.

**Figure 2:**
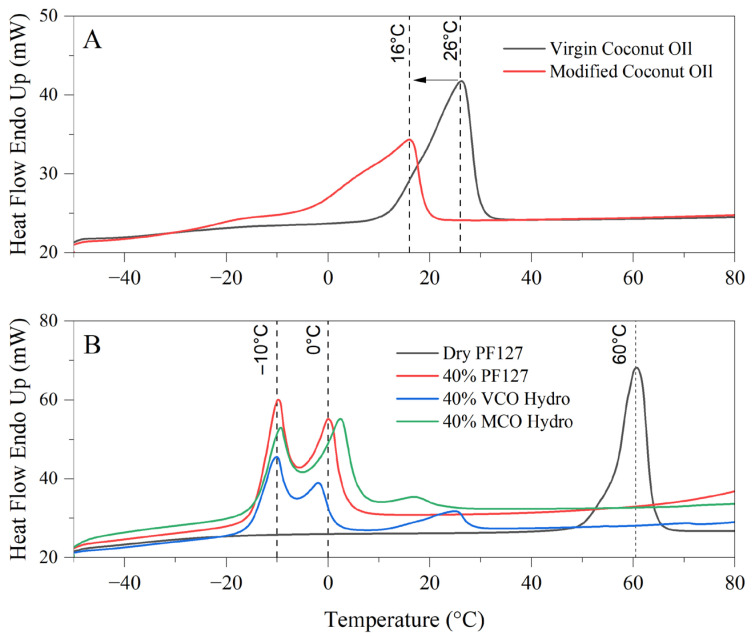
Thermograms of virgin and modified coconut oil (**A**) and bioactive PF127-Based Hydrogels (**B**) for thermal behaviour and formulation stability.

In Figure 2A, the melt profiles of VCO and MCO are compared to elucidate the effects of chemical modification on thermal behaviour. Virgin coconut oil exhibits a broad, reflecting the heterogeneous mixture of fatty acid triglycerides naturally present in unrefined coconut oil, which includes lauric, capric, caprylic, myristic, and other medium-chain fatty acids. The melting profile corresponds well with known polymorphic forms and the mixed acylglycerol content of VCO [18]. In contrast, the modified coconut oil demonstrates a sharper, more distinct endothermic peak centred around 16 °C. This shift to a lower temperature and the sharper peak suggests an increase in heterogeneity and decreased crystallinity, attributed to the introduction of polar functionalities as observed by FTIR in Figure 1. The increased breadth of the peak denotes a wider distribution of fatty acid species, implying that the MCO consists of a more heterogeneous composition. Additionally, the early onset of the VCO melting peak at approximately 26 °C compared to MCO highlights greater susceptibility to thermal softening in MCO, which can limit its performance in formulations exposed to warmer climates.

In Figure 2B, the thermograph depicts the melt behaviour of innate dry PF127, 40% PF127 hydrogel and the respective 40% VCO and 40% MCO hydrogels. Dry PF127 exhibited a sharp endothermic peak at 60 °C, corresponding to its crystalline melting temperature [19]. This peak is absent in the hydrated systems, indicating full dissolution and micellar dispersion of PF127 in aqueous environments, which is a prerequisite for hydrogel formation. In all hydrated systems, multiple transitions were observed in the sub-zero to ambient temperature range. Notably, the prominent endothermic peaks at −10 °C and 0 °C correspond to the melting of water within the hydrogel matrices. These peaks are characteristic of free and bound water fractions present in the hydrogel network [20]. The presence of these transitions confirms the retention of significant water content, a key indicator of hydrogel integrity and performance [21].

With respect to the bioactive hydrogels, the addition of VCO and MCO alters these thermal transitions, noting interactions between the oil phases and the micellar network of PF127. Compared to the innate 40% PF127 system, the incorporation of MCO resulted in broader pronounced thermal transitions, particularly in the 0 °C to 20 °C range, attributed to stronger interactions or improved miscibility between the MCO and the PF127 micellar structure. These interactions are possibly due to its more uniform composition and enhanced chemical compatibility. Conversely, the VCO hydrogel presents more dampened and less distinct thermal events, indicative of phase separation or less efficient incorporation of the unmodified oil into the hydrophilic polymer matrix.

### 2.3. Rheological Evaluation

#### 2.3.1. *Amplitude Sweep*

The deformation response of Pluronic hydrogels was assessed across the oscillation strain range from 0.01 to 100 rads/s. In accordance with the ISO 6721-10 [22] and EN/DIN EN 14770 [23] standards, it was determined that a 1% strain was sufficient for subsequent testing as the polymeric material deformed by less than 5%. As all formulations consisted predominantly of PF127, all bioactive hydrogels were also subjected to a 1.0% strain for subsequent testing.

#### 2.3.2. *Frequency Sweep*

**Figure 3:**
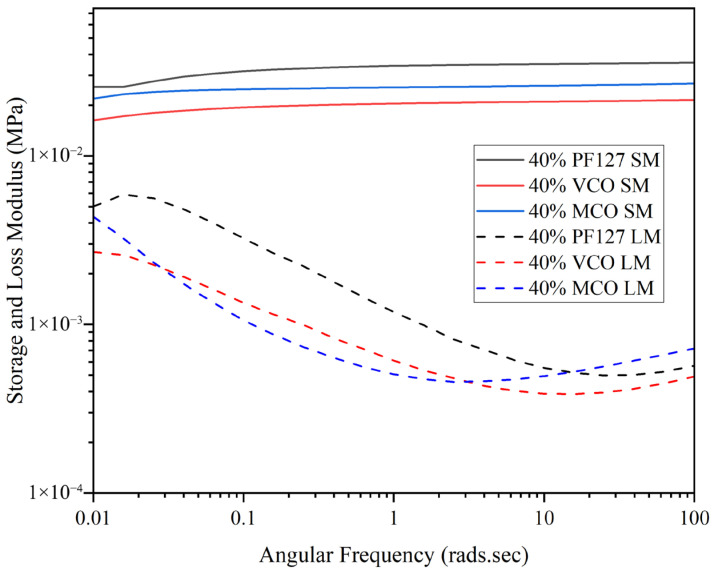
Frequency sweep of innate and formulated 40% hydrogels at 37 °C across an angular frequency range of 0.01–100 rads.s (*n* = 3).

The structure of hydrogels allows them to exhibit both elastic (solid-like) and viscous responses under varying frequencies of deformation. Evaluating these behaviours across a wide frequency range is essential, as many hydrogel systems display complex, frequency-dependent mechanical properties. As illustrated in Figure 3, all 40% formulations were observed to present dominance of the storage modulus (G′) over the loss modulus (G″) across the frequency range, denoting the systems were predominantly elastic and had completed the sol–gel transition at physiological temperature (37 °C).

The storage modulus of the PF127 hydrogel was consistently higher than those of the VCO and MCO formulations, with only a weak dependence on frequency as presented in Figure 3. This suggests strong structural stability of the PF127 matrix, a characteristic consistent with previous reports on Pluronic-based hydrogels at similar concentrations [24]. The incorporation of VCO and MCO into the PF127 matrix resulted in a minor reduction in storage modulus as compared to pure PF127, attributed to a degree of plasticisation due to the oil components. Despite this, both bioactive systems (VCO and MCO) maintained G′ > G″ across all frequencies, highlighting that the gels were consistently in the solid-like phase and were structurally stable under oscillatory deformation.

**Figure 4:**
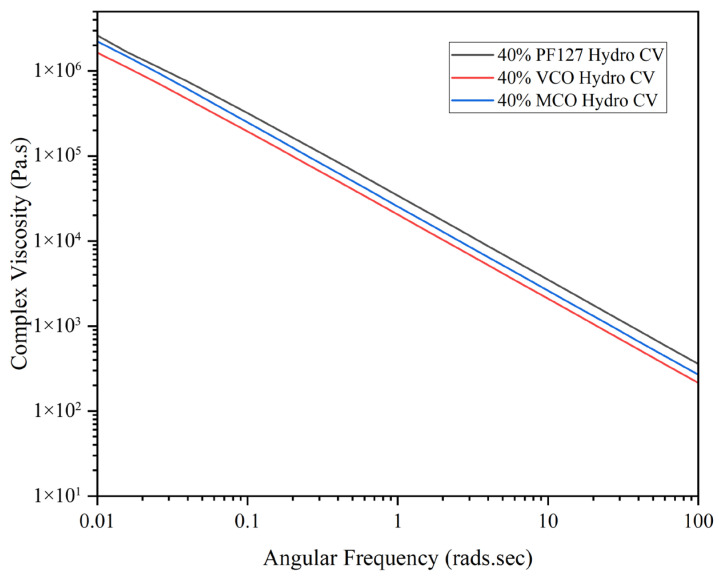
Complex viscosity of innate and formulated 40% hydrogels at 37 °C across an angular frequency range of 0.01–100 rads.s (*n* = 3).

As shown in Figure 4, all formulations displayed shear-thinning behaviour, with complex viscosity decreasing steadily as angular frequency increased. This pseudoplastic behaviour is characteristic of many hydrogel systems and is advantageous for biomedical applications such as injection, spreading, or topical delivery. Among the tested materials, PF127 maintained the highest complex viscosity across all frequencies, followed by MCO and VCO, attributed to the oil compounds reducing the network integrity by disrupting intermolecular interactions within the Pluronic structure [25]. This rheological behaviour marked by high elasticity at low strains and shear-thinning under increasing stress, makes these hydrogels suitable for applications requiring controlled delivery and mechanical resilience, such as injectable therapeutics or wound dressings. The data confirm that all three formulations are capable of withstanding mechanical stress while offering tuneable viscoelastic properties depending on the nature of the incorporated additive (VCO or MCO).

#### 2.3.3. *Temperature Sweep*

**Figure 5:**
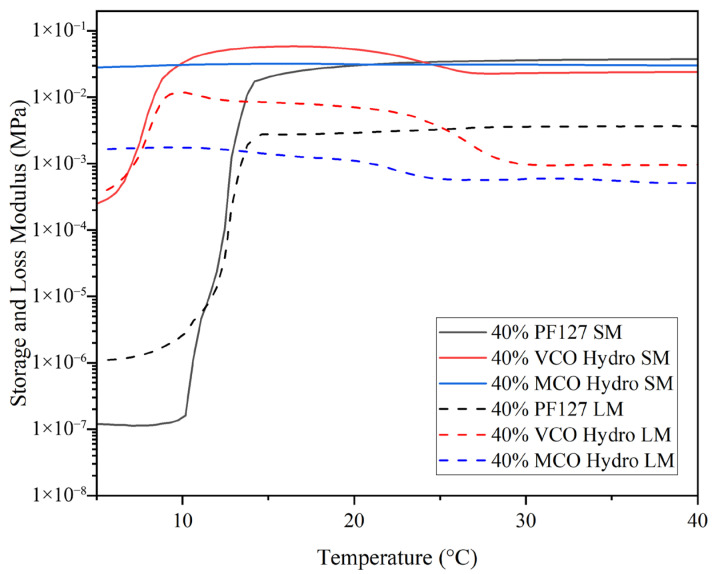
Temperature ramps of innate and formulated 40% hydrogels at 37 °C across a temperature range of 5 °C to 40 °C to determine gelation crossover point (*n* = 3).

Pluronic-based hydrogels are strongly influenced by polymer concentration; higher concentrations result in a lower gelation temperature. Investigating the influence of bioactive components on this transition allows tuning of hydrogel properties for biomedical use. For the purpose of this study, the sol–gel transition point was determined as the initial temperature at which the storage modulus (G′) exceeded the loss modulus (G″), signifying a transformation from a liquid-like (viscous) to a solid-like (elastic) behaviour. At lower temperatures in Figure 5, the innate 40% PF127 hydrogel was observed to present very low G′ and G″ values, denoting its viscous or sol-like state. As the temperature increased, a sharp rise in G′ occurred, overtaking G″ at ~11 °C, marking the sol–gel transition, signifying the conversion from liquid-like to a solid-like state, where elastic behaviour dominates. Following this transition, the storage modulus plateaued and remained consistently higher than the loss modulus, demonstrating that a stable, crosslinked gel network had formed. This pattern is characteristic of the micelle packing and network development expected in concentrated PF127 solutions, where temperature induces aggregation and physical gelation through the self-assembly of polymer micelles. The incorporation of virgin coconut oil (VCO) into the PF127 matrix led to a notable alteration in the hydrogel’s thermal response. The gelation point shifted to a much lower temperature, with G′ surpassing G″ at ~7.5 °C. This early transition suggests that the presence of VCO facilitates micellisation and network formation, through enhanced hydrophobic interactions between the oil phase and the hydrophobic polypropylene oxide segments of the PF127 copolymer. Unlike the sharp increase in G′ seen in the innate PF127 hydrogel, the VCO-containing formulation displayed a more gradual rise, indicating a broader gelation range. Once the gel state formed, the hydrogel’s storage modulus remained the highest among all three systems, implying that the presence of VCO contributed to a stiffer and more elastic network. In contrast, the incorporation of the MCO resulted in a subtler modification of the gelation profile. The MCO-loaded hydrogel exhibited an elevated storage modulus at lower temperatures, with G′ consistently higher than G″ throughout much of the temperature range. This denotes that the MCO formulation behaves more like a pre-structured gel even before typical gelation temperatures are reached. This result is supported by the work of Kjøniksen et al. (2014), in that the hydrophobic moieties need to be long enough to penetrate into the core of the Pluronic micelles, and together with the polymer backbone form bridges between the cores, thus inducing gel stabilisation [17].

Although the final storage modulus was not as high as that of the VCO hydrogel, it remained comparable to, or slightly greater than, the innate PF127 hydrogel, implying that MCO incorporation does not compromise mechanical integrity. The findings of Gwarzo et al. (2022) [26] note that the primary limitation of liquid dosage forms is their inability to retain the therapeutic agent at the wound site for a sufficient duration to achieve optimal therapeutic efficacy. These results support the use of Pluronic hydrogels as a delivery vector for coconut oil subtypes for greater retention at the site of fungal infection in contrast to the application of the oils alone [26].

### 2.4. Stability Testing

Formulated hydrogels were assessed for stability testing to assess their physical and microbial stability over time under ambient storage conditions as presented in Table 1. Innate Pluronic F127 hydrogels are known to lack antimicrobial characteristics. Post formulation (T_0_), both hydrogels exhibited a smooth, white, and homogeneous appearance, indicating successful emulsification and consistent formulation. There were no visible signs of discolouration, phase separation, or microbial contamination in either the VCO or MCO sample. The pH levels of both hydrogels ranged from 5.5 to 6.5, confirming their suitability for topical use at the outset. However, as the stability study progressed over the 28-day period, notable differences emerged between the VCO and MCO hydrogel samples.

The VCO hydrogels presented visible microbial contamination after 14 days, with mould colonies appearing on the surface. This microbial activity was accompanied by an unpleasant odour, suggesting rancidity and microbial decomposition of organic constituents. The texture of the hydrogel also deteriorated, becoming uneven and less cohesive. Evidence of phase separation became increasingly apparent, indicating the breakdown of the emulsion structure. After the 28-day period, the VCO hydrogels were visibly compromised, with extensive microbial growth covering the surface. The pH also showed a slight decrease, likely due to acidic metabolic by-products produced by the growing microorganisms. These findings indicate that the VCO hydrogel formulation lacked adequate preservation and was unable to maintain stability under ambient storage, rendering it unsuitable for long-term application without additional antimicrobial agents or refrigeration. In contrast, the MCO hydrogels demonstrated prominent stability throughout the study period. The samples maintained their original white, smooth appearance with no signs of microbial growth, discolouration, or separation. The odour remained consistent and pleasant, and the formulation did not exhibit any rancidity or spoilage. The texture and consistency of the MCO hydrogels remained intact, suggesting strong emulsion stability. Moreover, the pH remained stable over time, reflecting chemical resilience and an absence of microbial fermentation or degradation. These results suggest that the MCO formulation possessed improved antimicrobial and oxidative resistance, due to structural modifications in the coconut oil. The MCO hydrogel’s consistent performance across all tested parameters highlights their superior formulation stability in comparison to the VCO hydrogels.

After the 28-day period, Scanning Electron Microscopy (SEM) was conducted after the 28-day stability period, revealing the presence of microbial colonisation on the surface of the virgin coconut oil (VCO) hydrogels as presented in Table 2, while no such growth was observed on the modified coconut oil (MCO) hydrogels. In the VCO samples, microorganisms were evident on the external surface but were also embedded within the hydrogel matrix, suggesting active infiltration and proliferation throughout the formulation. The micrographs indicated dense clusters of microbial cells adhered to the hydrogel interface, with some appearing to penetrate the structural network, potentially facilitated by microphase separation or structural weaknesses inherent to the VCO formulation as noted in the DSC and rheology sections. This microbial infiltration likely correlates with the observed visual spoilage and surface degradation noted during the stability assessment, further emphasising the vulnerability of the VCO hydrogel to microbial contamination. In contrast, the MCO hydrogels displayed an intact and smooth surface morphology, with no detectable microbial presence, indicating superior resistance to microbial growth. The authors acknowledge that a longer stability assessment may be necessary for a pharmaceutical product, especially one intended for chronic conditions such as onychomycosis.

### 2.5. Well Diffusion Assay

To investigate the dermatological therapeutic potential of hydrogel formulations, the antifungal activity of innate oils and hydrogel formulations were assessed against *T. rubrum* and *C. albicans*; two dermatologically significant fungal species and presented in Table 3. The well diffusion assay presented consistent trends, with the modified coconut oil and the 40% MCO hydrogel demonstrating significant antifungal activity against both *T. rubrum* and *C. albicans*, whereas virgin coconut oil and the 40% VCO hydrogel consistently lacked the development of any zone of inhibition across all experiments. While assessing *C. albicans*, MCO alone produced the highest mean zone of inhibition (10.75 ± 0.35 mm), followed by MCO hydrogel (8.17 ± 0.76 mm). Similarly, the MCO formulations were effective against *T. rubrum*, with inhibition zones comparable to or exceeding those observed for *C. albicans*, highlighting their broad-spectrum antifungal efficacy. As innate Pluronic hydrogels are known to lack antimicrobial capacity, the antifungal effects are attributed to MCFAs alone and not synergistic interactions with PF127. The lack of activity observed in the VCO and VCO hydrogel underscores the importance of chemical modification in enhancing the functional performance of natural oils. Moreover, the incorporation of MCO into a 40% PF127 hydrogel not only retains antifungal activity but also offers potential advantages in terms of controlled release, improved stiffness and temperature stability for topical dermatological treatments. The authors acknowledge that the overall diversity of fungal pathogens implicated in onychomycosis is broader and thus, the study’s findings on antifungal efficacy are limited specifically to these two strains and may not be consistent across a full spectrum of fungal infections.

## 3. Conclusions

This study has demonstrated that the chemical modification of coconut oil significantly influences the structural, chemical, and antifungal properties of PF127-based hydrogels. FTIR analysis revealed that modified coconut oil (MCO) introduces additional polar functionalities, notably hydroxyl groups, enhancing its interaction with the polymer network compared to virgin coconut oil (VCO). Thermal analysis further showed that MCO alters the intrinsic melting behaviour of the oil and improves compatibility with the thermoresponsive polymer system, resulting in enhanced gel uniformity, stability, and mechanical integrity across a broad temperature range. Both VCO and MCO hydrogels modified the thermal gelation behaviour of PF127 in distinct ways, offering tailored mechanical responses. Importantly, MCO hydrogels exhibited superior formulation stability and consistent performance across multiple parameters compared to VCO hydrogels, which showed limited activity. This is important as it addresses the inability of liquid dosage forms to retain the therapeutic agent at the infection site due to low viscosity and temperature sensitivity. These results support the use of Pluronic hydrogels as a delivery vector for coconut oil subtypes for greater retention at the site of fungal infection in contrast to the application of the oils alone. The incorporation of MCO into a 40% PF127 hydrogel not only preserved antifungal activity but also provided potential advantages such as controlled release, improved stiffness, and enhanced temperature stability, making it especially promising for topical dermatological treatments. These findings highlight the critical role of chemical modification in enhancing the functional performance of natural oils within hydrogel systems and provide a strong foundation for further exploration of their biomedical applications in the treatment of onychomycosis.

## 4. Materials and Methods

### 4.1. Materials

Virgin coconut oil (VCO) was purchased as a representative of innate coconut oil on the market. Modified coconut oil (MCO) was prepared via a proprietary procedure developed at the Technological University of the Shannon (TUS), the affiliation of the corresponding authors. These materials were stored at room temperature (20 °C ± 2 °C) throughout testing period. Pluronic F127 (PF127) was purchased from Sigma-Aldrich Ireland Ltd. (Arklow, Wicklow, Ireland). Ultrapure water (ASTM-1) was obtained from a Barnsted Smart Pure Pro water purification system (Thermo Fisher Scientific, Waltham, MA, USA). All materials were used as received.

### 4.2. Hydrogel Preperation

Pluronic F127 hydrogels at 50% concentration (*w*/*v*) were prepared using the cold method described by Schmolka (1972) with an adaptation to account for sterilisation [27]. The flask was subsequently placed on ice and routinely shaken to ensure solubilisation of the Pluronic material and formation of the hydrogel system [19]. These formulations were subsequently diluted with virgin and modified coconut oil to reach the desired (40%) final concentration as presented in Table 4.

### 4.3. Preparation of Bioactive Hydrogel

Bioactive hydrogels were produced by physically mixing cold (4 °C) 50% PF127 hydrogels with virgin coconut oil (VCO) and modified coconut oil (MCO), in their liquid phase, to reach a final polymer concentration range of 40% PF127. All formulations were physically mixed in a small mortar and pestle until the mixture reached room temperature to ensure homogeneous texture. Samples were stored at room temperature until required for analysis.

### 4.4. Attenuated Total Reflectance Fourier Transform Infrared Spectroscopy (ATR-FTIR)

The spectra of the samples were performed on a spectrophotometer ATR-FTIR (PerkinElmer Spectrum One with a universal ATR sampling accessory, Shelton, Connecticut, United States). They were scanned between 650 and 4000 cm^−1^, on average 16 scans per sample, and a fixed universal compression force of 80 N. Further analyses were carried out using Origin 2023b software.

### 4.5. Differential Scanning Calorimetry

Thermal characterisation of innate materials and hydrogel formulations (10 mg–15 mg) were analysed using DSC (PerkinElmer Pyris 1) at a scanning rate of 10°C min^−1^ with a single heating and cooling run from −50–80 °C. Pyris data analysis (Version 13) and Origin 2023b software was used to process the raw datasets.

### 4.6. Rheological Evaluation

Rheological properties of all samples were assessed on a DHR20 rheometer (TA Instruments, DE, USA) with a refrigerated cooling system (RCS120) and TRIOS software (Version: V4.1.1.33073) for data analysis. A 50 mm stainless steel geometry was utilised in adjacent to a Peltier plate. All samples were loaded in their respective cold phase with either a spatula or as 1.0 mL volumes using a 3 mL Pasteur pipette. A soak time of sixty seconds was warranted prior to data acquisition to stabilise environmental conditions and remove any interfering stress induced during sample loading. After equilibration, quintuplet amplitude sweeps were conducted to determine the linear viscoelastic region (LVER) of individual samples in order to select an appropriate oscillation stress (1%) at which to conduct further rheological measurements. The storage modulus was measured as the samples were subjected to oscillation strain that steadily increased from 0.01% to 100% at an oscillation frequency of 6.28 rad/s. The LVER of each sample was reported as the range of oscillatory strain for which the observed storage modulus of the material changed by less than 5% with subsequent measurement in accordance with the ISO 6721-10 and EN/DIN EN 14770 standards.

#### 4.6.1. *Frequency Sweep*

Oscillation frequency sweeps were conducted at 37 °C over an angular frequency of 0.1 to 100 rad/s under a constant strain of 1.0%.

#### 4.6.2. *Temperature Sweep*

Temperature ramps were conducted to observe the sol–gel phase transition of the hydrogels across a temperature range from 4 to 40 °C under a constant strain of 1.0%, as determined from the amplitude sweep.

### 4.7. Stability Testing

Stability testing at room temperature (RT) for the cream product was conducted in a controlled laboratory environment to assess the product’s physical, chemical, and microbiological stability over time. The cream samples were stored at RT (typically 25 ± 2 °C/60 ± 5% RH) in their final packaging materials. At predefined intervals (0, 14, and 28 days), the samples were evaluated for changes in appearance, colour, odour, phase separation, pH and the presence of microbes.

### 4.8. Scanning Electron Microscopy

The SEM/EDX Analysis was performed using a TESCAN SEM (JEOL (Europe) BV, Haarlemmermeer, The Netherlands) with an EDX Detector for microbial imaging. Specimens were mounted onto aluminium sample stubs coated with a surface adhesive to hold them in place. Samples were sputter coated in gold prior to SEM imaging.

### 4.9. Antimicrobial Screening via EUCAST Well Diffusion Assay

Two panels of fungal strains were used for antimicrobial testing and validation: *Candida albicans* (ATCC 10231) and *Trichophyton rubrum* (ATCC 28188). Lyophilised cultures were acquired from their respective culture collection institutes. Primary fungal strains were preserved at −80 °C on Cryobeads™ (Copan Diagnostics, Murrieta, CA, USA) and glycerol stock at 20%. Antimicrobial work was conducted in a biosafety cabinet as *T. rubrum* is categorised as a biosafety level 2 organism. Fungal densities were obtained by spectrophotometry to ensure reproducible inoculation for testing purposes.

The microorganisms were cultured in Sabaroud dextrose broth (SDB) and Sabaroud dextrose agar (SDA) (Accumedia lab NEOGEN culture media; Cruinn Diagnostics, Dublin, Ireland). *Candida albicans* (ATCC 10231) was cultured at 37 °C for 2 days aerobically. Microbial suspensions were prepared by inoculating microbial colonies from the isolate culture into 20 mL of Sabaroud dextrose broth and incubated at 37 °C for 24 h aerobically without shaking. *Trichophyton rubrum* (ATCC 28188) was cultured at 30 °C for 15 days to isolate conidia and induce full sporulation.

To conduct the well diffusion assay, SDA plates were prepared with 1 mL of fungal inoculum in Petri dishes. A sterile well puncher was used to create four wells of approximately 9 mm in the agar plates with even spacing. Samples were added into the wells at a volume of 200 µL using sterile pipettes. Plates of *Candida albicans* were sealed with parafilm, inverted, and placed in a 37 °C incubator for 2 days, whereas plates inoculated with *Trichophyton rubrum* were sealed with parafilm, inverted, and placed in a 30 °C incubator for 5–7 days. After incubation, the plates were visually inspected for zones of inhibition around the wells and measured to the nearest millimetre.

## Figures and Tables

**Table 1:** Stability testing of 40% coconut oil hydrogels for changes in appearance, colour, odour, phase separation, pH and the presence of microbes.

	40% VCO	40% MCO
T0	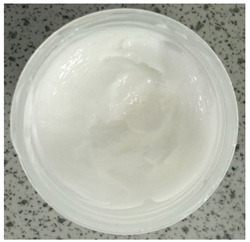	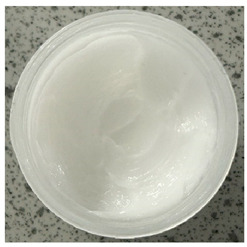
T14	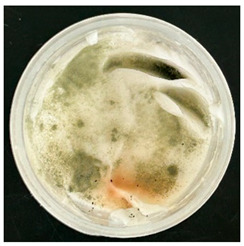	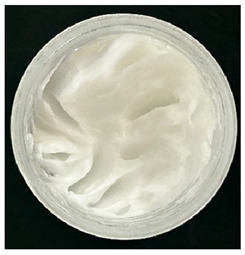
T28	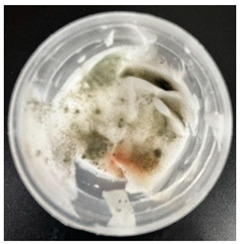	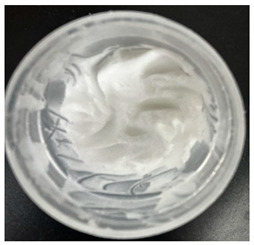

**Table 2:** SEM images of 40% VCO and MCO hydrogels after stability testing at room temperature for 28 days.

Magnification	40% Virgin Coconut Oil Hydrogel	40% Modified Coconut Oil Hydrogel
200×	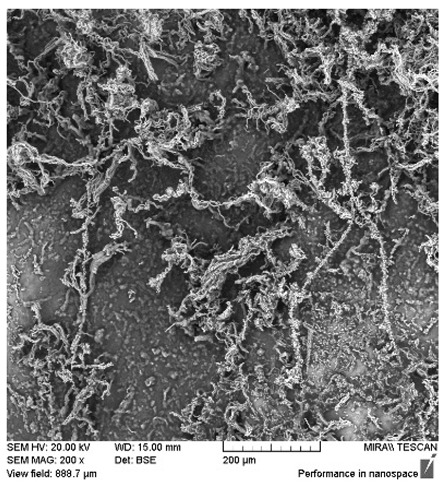	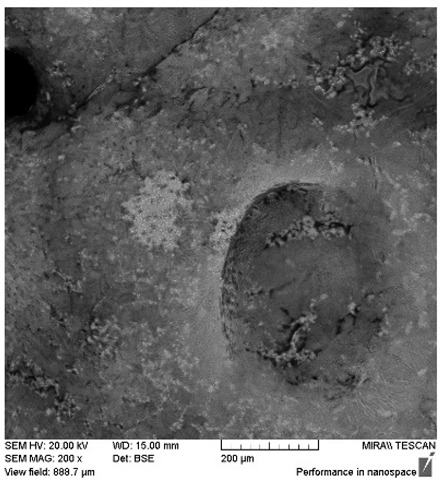
500×	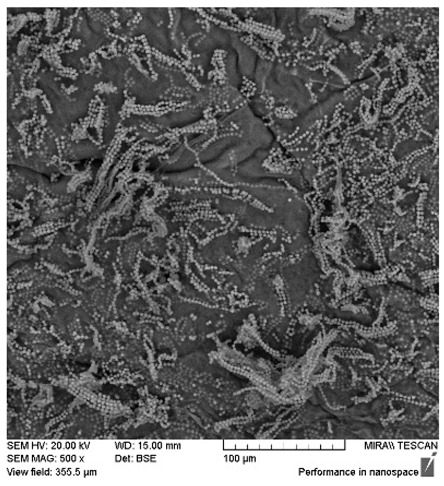	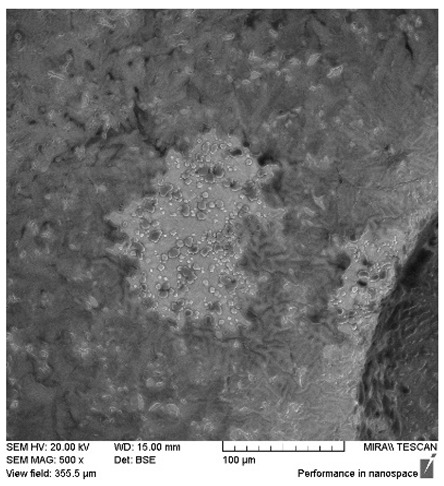
1000×	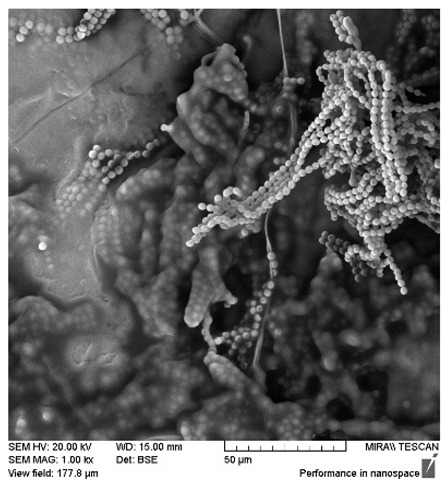	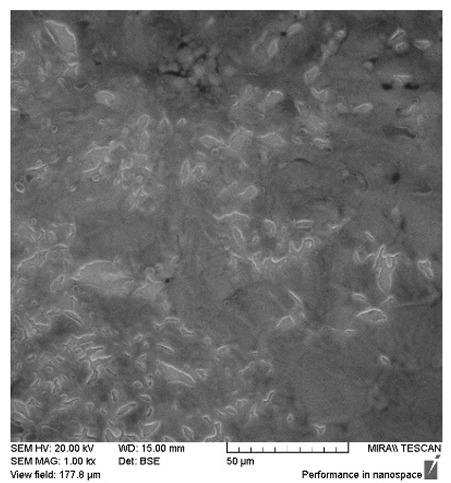

**Table 3:** In vitro antifungal assessment of virgin and modified coconut oils and their respective hydrogel formulations targeting *T. rubrum* and *C. albicans*.

	Zone of Inhibition Diameter (mm)	
Sample of Interest	*C. albicans*	*T. rubrum*
VCO	0	0
MCO	10.75 ± 0.35	10.75 ± 0.35
40% PF127	0	0
40% MCO Hydrogel	8.17 ± 0.76	11.6 ± 1.5
40% VCO Hydrogel	0	0

**Table 4:** Formulations of high concentration PF127 and the respective coconut oils.

	PF127 (mL)	VCO (mL)	MCO (mL)	Final [PF127] (%)	Final [CO] (%)
40% PF127 (Control)	16 *	-	-	40 *	-
40% VCOH	16 *	4	-	40	20
40% MCOH	16 *	-	4	40	20

* Pluronic hydrogel at 50% concentration was used to produce a final polymer concentration of 40% with ddH_2_O as the diluent.

## Data Availability

The raw data supporting the conclusions of this article will be made available by the authors on request.
